# Binding of more than one Tva800 molecule is required for ASLV-A entry

**DOI:** 10.1186/1742-4690-8-96

**Published:** 2011-11-18

**Authors:** Eleanor R Gray, Christopher JR Illingworth, John M Coffin, Jonathan P Stoye

**Affiliations:** 1 Division of Virology, MRC National Institute for Medical Research, The Ridgeway, Mill Hill, London NW7 1AA, UK; 2 Wellcome Trust Sanger Institute, Wellcome Trust Genome Campus, Hinxton, Cambridge, CB10 1SA, UK; 3 Department of Molecular Biology and Microbiology, Tufts University School of Medicine, 150 Harrison Avenue, Boston, MA02111, USA; 4 Current Address: Dept of Infection and Immunity, University College London, Cruciform Building, Gower Street, London WC1E 6BT, UK

## Abstract

**Background:**

Understanding the mechanism by which viruses enter their target cell is an essential part of understanding their infectious cycle. Previous studies have focussed on the multiplicity of viral envelope proteins that need to bind to their cognate receptor to initiate entry. Avian sarcoma and leukosis virus Envelope protein (ASLV Env) mediates entry via a receptor, Tva, which can be attached to the cell surface either by a phospholipid anchor (Tva800) or a transmembrane domain (Tva950). In these studies, we have now investigated the number of target receptors necessary for entry of ASLV Env-pseudotyped virions.

**Results:**

Using titration and modelling experiments we provide evidence that binding of more than one receptor, probably two, is needed for entry of virions via Tva800. However, binding of just one Tva950 receptor is sufficient for successful entry.

**Conclusions:**

The different modes of attachment of Tva800 and Tva950 to the cell membrane have important implications for the utilisation of these proteins as receptors for viral binding and/or uptake.

## Background

Entry of a retrovirus into a cell represents one of the most important steps in the viral life cycle. The virus must first target cells expressing the appropriate receptor, and once bound, overcome the energetic barrier presented by the plasma membrane to enter the cell. Within the family of *Retroviridae*, there are many variations in receptor type and usage. The nature of the interaction between the retroviral envelope protein and its cognate receptor and coreceptor (if used), are key to understanding the process of viral entry. To date, most *in vitro *and *in silico *studies of the quantitative aspects of the interaction between trimeric viral envelope (Env) protein and its cognate receptor have focussed on HIV-1 and CD4 e.g. [1-5]. However, these are complicated by the necessary interaction of the virus with both receptor and coreceptor and the interdependence of CD4, CXCR4 and CCR5 levels [6-9].

The alpharetrovirus ASLV-A can utilise one of two receptors for entry, Tva800 or Tva950, which arise from alternate splicing of a single gene [10,11]. Both comprise identical 83-amino acid binding domains with an LDL-A motif [11], but differ in their attachment to the membrane. Tva800 has a C-terminal GPI anchor sequence, but Tva950 is attached by a single transmembrane spanning domain. As a result, the receptors are localized to different regions of the cell membrane; Tva800 is found in lipid rafts, whereas Tva950 is excluded from these areas [12]. The normal cellular functions of Tva800 and Tva950 are not known.

Fusion mediated by ASLV Env and Tva800 or Tva950 requires a low pH step for entry via endosomes [13,14]. Virus binding triggers a conformational change in Env that exposes the fusion peptide, which is then inserted into the cellular membrane [13,15]. Exposure of the sensitised Env to low pH in the endosomes will lead to fusion of viral and cellular membranes [16]. Tva800 and Tva950 possess the same N-terminal Env-binding and fusion-mediating domain, but the kinetics of entry differ, as Tva800 mediates the process more slowly [17,18].

For the present study, examination of the interaction between ASLV-A Env and its two receptors was initiated to investigate how the difference in attachment might affect subsequent movement of ALSV-pseudotyped MLV into sub-cellular compartments, with corresponding differences in susceptibility to the cellular restriction factors TRIM5α [19] and Fv1 [20]. However, initial experiments revealed that even before the virus enters the cell, the difference in membrane attachment of the two receptors can affect virus uptake. The chance isolation of a transducing vector expressing very low levels of receptor presented the opportunity for the investigation of the stoichiometric requirements for the two forms of the receptor. Here, we present data that are the counterpart of studies on the number of Env trimers that must be bound for entry, namely the number of receptors that must be bound. We found that binding of more than one Tva800 is required for a virus to be able to enter a cell, but that binding of a single Tva950 molecule is sufficient for entry. A possible explanation for this difference is proposed.

## Results

### Generation of Tva800-positive cell populations

**Figure 1:**
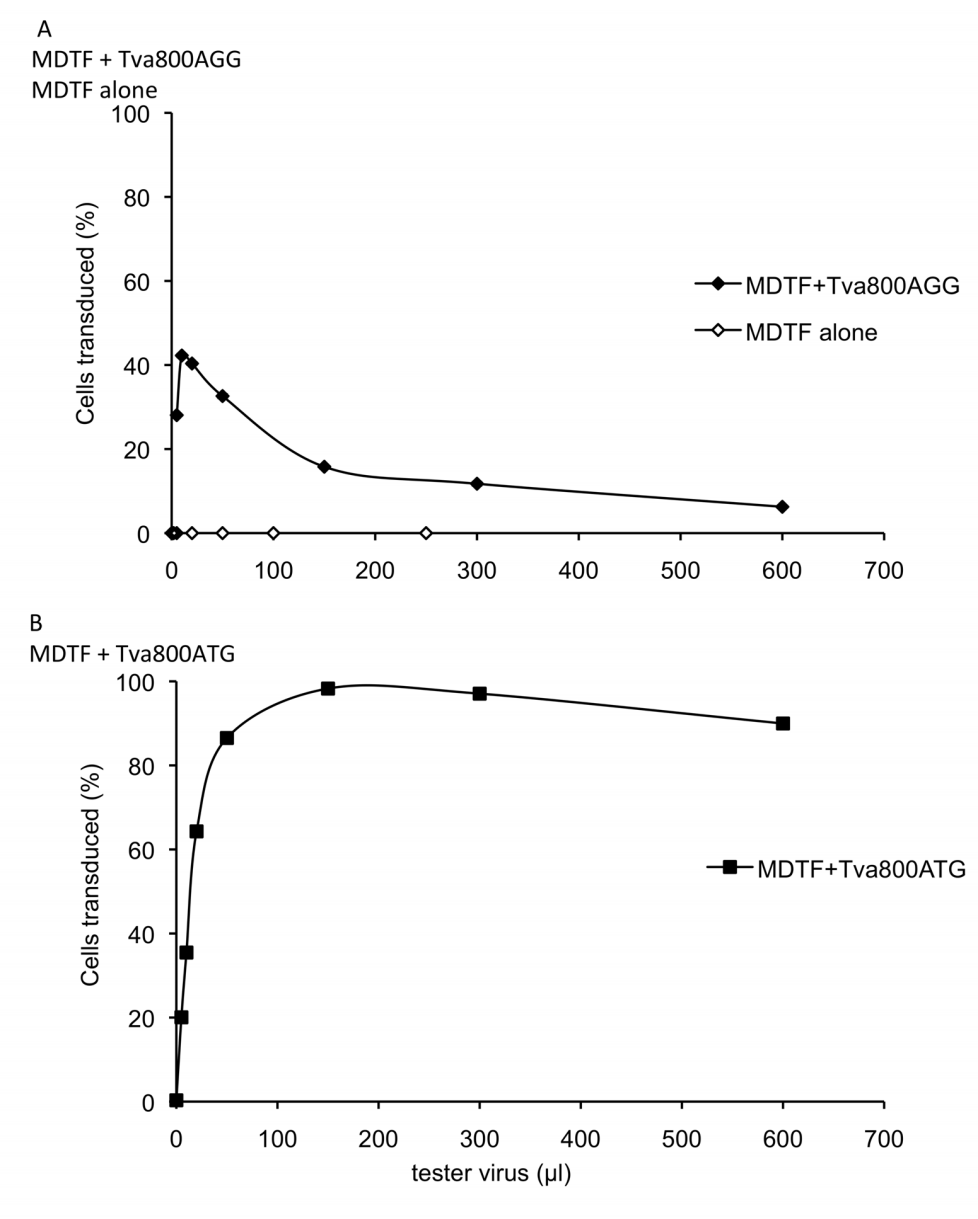
**Titration of ASLV-A Env pseudotyped NB-MLV on MDTF cells transduced with Tva800AGG or Tva800ATG**. Cells were initially transduced at an MOI of approximately 1 i.u./cell with a delivery virus expressing Tva800 and YFP and challenged 3 days later with 5-600 μl ASLV-A pseudotyped NB-MLV expressing GFP (tester virus). Cells were analyzed by two-colour FACS after a further three days. Data are plotted as the percentage of delivery virus transduced cells (YFP positive) infected by tester virus (GFP positive). (A) Mock-transduced (open circles) and Tva800AGG (filled circles). (B) Tva800ATG-transduced. Shown are the combined results of two independent experiments for (A) and one experiment for (B).

Initially intending to investigate the effect of different uptake pathways on restriction by TRIM5 and Fv1, we set out to prepare MDTF cells carrying receptors for a number of orthoretroviral Env proteins, among them Tva800. To construct one such cell line, the *Tva800 *gene was cloned into the vector pLgatewayIY [21]. Transduction of MDTF cells with one particular clone of this construct at MOI 1 infectious unit (i.u.)/cell yielded a polyclonal population of cells in which YFP-positive cells can reasonably be expected to express Tva800. When these cells were challenged with ASLV-A Env-pseudotyped NB-MLV, the titration curve shown in Figure 1A was obtained. This curve was not of the expected shape; when more than 10 μl of tester virus was added, the percentage of infected cells did not continue increasing as the volume of challenge virus increased. The same curve was seen in two repeat experiments. When the Tva800-YFP clone was sequenced a single difference to the published sequence for Tva800 (GenBank ID 403161) was identified. Instead of the translational start codon of ATG, a codon of AGG was found. It would be expected that this would not result in any protein production when this vector was transduced into cells. However, MDTF cells do not harbour an endogenous receptor for ASLV-A Env-pseudotyped virus, and proved to be completely non-permissive when challenged without initial transduction of the Tva800 vector (Figure 1A), implying some translational activity of the mutant gene.

To test whether the T to G change was responsible for the peculiar shape of the titration curves, the start codon was restored by a point mutation of G to T, and the titration curve was repeated, as shown in Figure 1B. Cells were transduced with this second Tva800 vector and then challenged with increasing amounts of ASLV-A Env pseudotyped virus. The percentage of infected cells rose rapidly and reached a plateau with nearly all cells infected (Figure 1B). These data would suggest that the mutant start codon was directly responsible for the atypical titration curve seen with the first vector. The two Tva800 vectors were denoted Tva800ATG and Tva800AGG, according to their start codon sequence.

### Protein analysis of Tva800 in cells transduced with HA-Tva800ATG or HA-Tva800AGG

**Figure 2:**
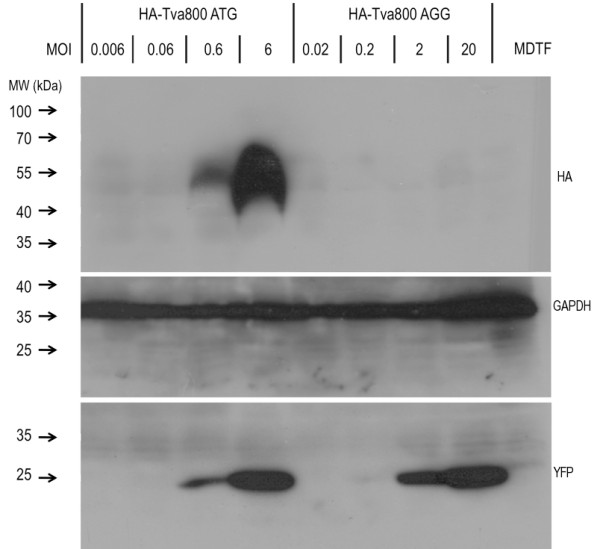
**Analysis of Tva800 expression in transduced cells**. MDTF cells were seeded in 60 mm dishes and transduced at the MOI (i.u./cell) shown with HA-Tva800ATG or HA-Tva800AGG. Cells were grown to confluence over 3 days, lysed with loading buffer, denatured and separated on a gel and examined by Western blotting, probing with firstly anti-HA, and secondly anti-GFP on the same gel. The blot was stripped and probed for GAPDH to check for comparable input lysate levels. Markers for molecular weight are shown on the left in kDa.

Given that Tva800 production has to take place to permit entry of ASLV-A Env-pseudotyped challenge virus into these cells, but that it is translated from a vector with an altered start site, it seemed likely that only a limited level of protein production occurred in these cells. This proposition was tested by Western blot analysis. First, a 27-bp sequence encoding an HA-tag was inserted into the expression vectors just after the signal peptide between residues 19 and 20 of Tva800. This 9 amino acid insertion did not have any effect on viral infectivity of the two new vectors, HA-Tva800ATG or HA-Tva800AGG (data not shown). Then, MDTF cells were transduced with HA-Tva800ATG at MOIs from 0.006 to 6, or HA-Tva800AGG at MOIs from 0.02 to 20i.u./cell. Proteins in cell lysates were separated by polyacrylamide gel electrophoresis, and analyzed after transfer by probing with either anti-HA, anti-YFP or, as loading control, anti-GAPDH (Figure 2). No HA-tag or YFP expression was detected with untransduced MDTF extract. As expected, no difference was seen in YFP expression with the two vectors, consistent with YFP production from the IRES being unaffected by the Tva800 start codon. By contrast, when probing for the HA-tag, bands were seen in the cells transduced with the HA-Tva800ATG vector at MOIs of 0.6 or above. This band had a somewhat lower mobility than expected from its amino acid content, presumably as a result of N-linked glycosylation at 3 sites [11]. Cells transduced with HA-Tva800AGG did not produce detectable levels of receptor protein, even when transduced at MOI 20 i.u./cell, despite producing significant levels of YFP protein at MOI 2 and 20 i.u./cell These data show that the level of Tva800 production from HA-Tva800AGG transduced cells is at most 30-fold lower than that from cells transduced with HA-Tva800ATG and likely substantially less.

### Increasing Tva800AGG normalises challenge virus titration curves

**Figure 3:**
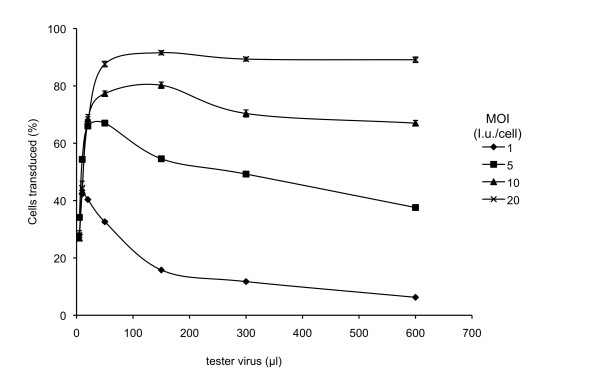
**Tester virus titration in cells transduced with Tva800AGG at different multiplicities**. MDTF cells transduced with Tva800AGG at MOI 1, 5, 10 or 20 i.u./cell were challenged with 5-600 μl ASLV-A Env pseudotyped NB-MLV and analyzed by two-colour FACS as described in the legend to Figure 1. The combined results of two independent experiments are shown. Error bars represent standard deviation of the mean.

If the low levels of Tva800 at the surface of the transfected cells are indeed responsible for the abnormal titration curves of cells transduced with Tva800AGG, transduction of cells at higher MOI should result in increased numbers of proviruses per cell leading to higher levels of Tva800 transcription and thus to higher levels of protein expression, potentially converting the curve shown in Figure 1A to that seen in Figure 1B. To test this idea, MDTF cells were transduced with Tva800AGG at MOI 1, 5, 10 and 20, then challenged with between 2-600 μl of tester virus. As the MOI at which cells were transduced with Tva800AGG increased, the resulting titration curves show shallower decreases in the number of cells infected with increasing tester virus dose (Figure 3). Increasing the MOI to 20i.u./cell resulted in a titration curve of the challenge virus that was essentially indistinguishable from that produced on challenge of cells transduced with Tva800ATG. This result is consistent with the hypothesis that low Tva800 receptor levels are responsible for the abnormal shape of the titration curves.

### Competition between SUA-rIgG and ASLV-A Env pseudotyped GFP vector virus

If the effective concentration of Tva800 on Tva800AGG-transduced cells is limiting, then infection of these cells should be particularly sensitive to competition with free receptor. To test the effect on viral titre mediated by increased blocking of free receptor, a constant volume of virions carrying GFP vector was titrated onto cells in competition with increasing volumes of SUA-rIgG. SUA-rIgG is a hybrid protein comprising the SU of ASLV-A Env linked to rabbit immunoglobulin G that can bind to receptors through the SU portion, and so block binding of GFP vector virus to Tva800. This experiment can directly indicate whether cells transduced with Tva800AGG express significantly less receptor than cells transduced with Tva800ATG, and the fraction of the GFP virus preparation composed of empty virions remains constant and can be ignored.

**Figure 4:**
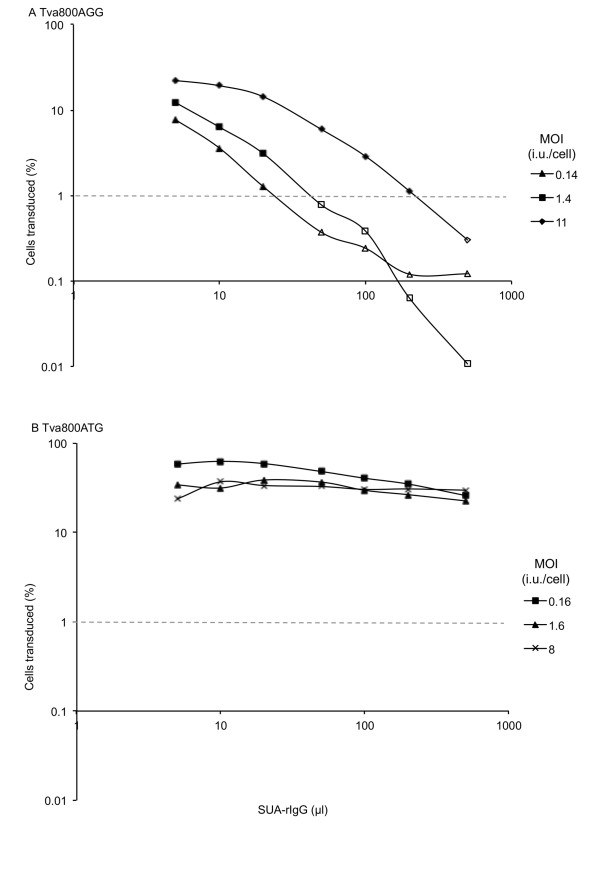
**SUA-rIgG competition of ALSV-A Env mediated infection**. Cells transduced with Tva800 vectors were challenged with 10 μl ASLV-A Env pseudotyped NB-MLV mixed with 0-500 μl of SUA-rIgG-containing supernatant,. Infection was assessed by two-colour FACS as described above. (A) Tva800AGG, MOI 0.14, 1.4 and 11. (B) Tva800ATG, MOI 0.16, 1.6, and 8. Representative results from three (A) and four (B) experiments performed are shown.

SUA-rIgG was able to compete with ASLV Env-pseudotyped virions as shown in Figure 4. Doubling the amount of SUA-rIgG in the challenge dose reduced the percentage of cells infected by half in cells transduced with Tva800AGG (Figure 4A), but no decrease was observed in the proportion of virions entering the cells transduced with Tva800ATG at MOI 1.6 or 8 (Figure 4B). There is a lag in decline of infectivity in cells transduced at MOI 11 with Tva800AGG presumably because these cells express sufficient receptor to sustain a small increase in blocking agent. In cells transduced with the lowest level of Tva800ATG, at MOI 0.16 (Figure 4B), a decrease of just over half of the maximum (59% to 26%) was seen. However, if this decrease is compared to cells transduced with Tva800AGG at a similar MOI (0.14), a decrease from a maximum of 7.8% cells GFP positive to less than 1% is seen. The final four data points show levels of GFP-positive cells that are below the limit of detection, suggesting that the fold-decrease might be much greater than 7.8. These data would support those shown in Figure 2, and the hypothesis that cells transduced with Tva800AGG produce significantly less Tva800 than cells transduced with Tva800ATG.

### Similar effects are not seen with Tva950

As discussed above, the ALSV-A receptor is found in two forms, Tva800 and Tva950. They are identical in the extracellular domain, including the viral envelope binding motifs, but differ in their attachment to cells [11]. Both are composed of identical 83-amino acid sections, after which Tva950 has a membrane-spanning domain, and Tva800 has a C-terminal GPI anchor signal sequence, and, after undergoing post-translational modification, will be attached to the lipid membrane via a GPI anchor. To assess whether the unusual titration curves obtained with low levels of Tva800 could be related to its attachment to the cell membrane, a comparison of the effects of low Tva950 production on the permissivity of cells to increasing doses of ASLV-A Env virions was performed. Tva950 was cloned into pLGatewayIY, after which the start codon of the resulting vector was mutated to AGG. The two vectors were named Tva950ATG and Tva950AGG respectively. They are identical to the corresponding Tva800 plasmids other than the 149 nucleotide insertion near the 3' end of the ORF that distinguish the two forms of the gene.

**Figure 5:**
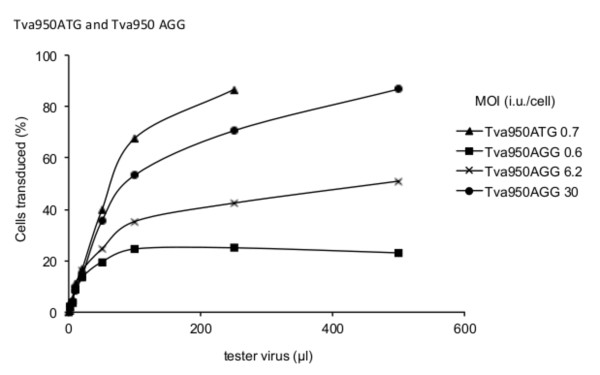
**Titration of ASLV-A Env pseudotyped NB-MLV on MDTF cells transduced with Tva950AGG or Tva950ATG**. Cells transduced with the levels shown of Tva950ATG or Tva950AGG (i.u./cell) were challenged with 5-500 μl ASLV-A Env pseudotyped NB-MLV and analyzed by two-colour FACS as described in the legend to Figure 1.

Figure 5 shows data from an experiment using these vectors to introduce the Tva950 receptor. Tva950 appears to be utilized less efficiently than Tva800 as judged by the shallower slope with the wild type form (compare Figure 1B with Figure 5). Cells transduced with Tva950AGG are less susceptible to infection than Tva950ATG; this effect can be overcome by transduction with higher MOI (Figure 5). However, in contrast to Tva800, there was no evidence for an inhibitory effect when high concentrations of tester virus were applied to cells presumed to carry low concentrations of receptor (i.e. transduced with Tva950AGG).

### Modelling the effects of limiting receptor on virus entry

We hypothesized that the limiting factor for viral entry might be free receptor and that virus must bind more than one receptor molecule to enter a cell. The low levels of Tva800 at the surface of cells transduced with low MOI of Tva800AGG would thus lead to reduced amounts of free, unbound receptor that become limiting for viral uptake with increasing doses of the tester virus. Consequently, with increasing virus challenge, more virions (bound only to one receptor) fail to enter the cell, either remaining at the cell surface or internalized but not fused, and the number of cells infected decreases.

**Figure 6:**
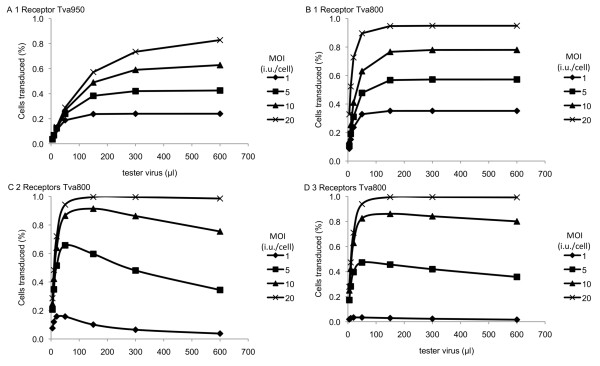
**Relationship between efficiency of ASLV-A Env mediated virus entry and the number of Tva800 or Tva950 receptors needed for entry**. The relative levels of receptor positive cells infected (% cells transduced, Y-axis) when titrating virus (μl, X-axis) onto cells previously transduced with different amounts of a vector encoding Tva800 or Tva950. Graph (a) shows the model for Tva950 when 1 receptor is needed, (b)-(d) assume binding of 1-3 Tva800 receptors, respectively. Data from the multiple-receptor model is not shown for Tva950. We note that the one-receptor model is a special case of the multiple-receptor model (the latter will approach the former as gamma tends to infinity). As the fit for the one-receptor model is very good, any results for larger numbers of receptors would be extremely close to those described here.

Application of a mathematical model gave an explanation for these results in terms of physical parameters. The use of differential equations and probabilistic methods to mathematically model viral systems is well established [22-24], and here a range of standard mathematical techniques were applied to simulate the various stages of the experiment. Full details of the model are given in additional file 1. Results from the model, and for the experimental data, are shown in Figure 6 and Table 1. The results for Tva950 suggest that this system is well characterised by a model in which a single receptor is required for the tester virus to gain entry to the cell, in which the efficiency of the delivery vector is high, but the receptor efficiency is low. In the case where the tester virus needs to bind a single receptor to gain entry to the cell, increasing the MOI of the tester virus means that there are more viruses available to bind receptors, which increases the proportion of cells that are successfully transduced with the tester virus. This consistent increase is observed in the data for Tva950 (compare Figure 6A with Figure 5).

**Table 1:** Optimised parameters from the mathematical model describing the proportion of MDTF cells expressing EYFP that also expressed EGFP

System	Number of receptors needed for viral entry *k*	Model error *D*	Efficiency of delivery vector *p*	Number of receptors produced for each TVA integration *r*	Receptor efficiency *q*	Relative rate at which bound viruses acquire more receptors *γ*
Tva950	1	0.0258	1	1.579	0.198	n/a

Tva800	1	0.106	0.25	0.921	1	n/a

Tva800	1	0.176	1*	0.925	0.300	n/a

Tva800	2	0.120	1	0.979	0.992	9.837

Tva800	3	0.165	1	0.990	1	159.3

In each case the number of receptors needed for viral entry was fixed, while other parameters were optimised to maximise the fit to the experimental data. A calculation for Tva800 with one receptor needed for viral entry was rerun, with the efficiency of the delivery vector fixed to match that obtained for Tva950. The error D gives the RMSD difference between the model and the experimental data in each case. Efficiencies are expressed as probabilities, such that an efficiency of 1 is equivalent to 100%.

*parameter fixed, not optimised

By contrast, the data with Tva800 suggest that this system is best characterised by a model in which more than one receptor is needed for the tester virus to gain entry to the cell (Figure 6B-D). Whereas, in the one receptor model, increasing the tester virus MOI always increases the likelihood of receptors being bound by viruses, and hence increases EGFP expression, this is not the case where more receptors are needed for viral entry. In the latter case, a competitive process occurs, in which viruses bound to receptors on the cell gradually accumulate more receptors, up to the number required for viral entry, while viruses in solution bind free receptors on the cell. In this case, an initial increase in the MOI of the tester virus increases the number of viruses available to bind receptors, increasing the proportion of cells expressing EGFP. However, when the MOI of the tester virus becomes large, the rate of binding of free receptors by virions in solution overtakes the rate at which virions already bound to the cell acquire additional receptors. Virions binding fewer than the required number of receptors for entry can occupy all of the receptors before a single virus binds enough of them to gain entry to the cell, so the proportion of cells expressing EGFP decreases. This behaviour, characterised by an initially increasing proportion of cells expressing EGFP followed by a decline at high tester virus MOI, is observed in the experimental data for Tva800.

Table 1 shows optimised parameters for each of the models. For the Tva950 receptor, a model in which a single receptor was required for viral entry gave a good fit to the experimental data. We note that the model systems for Tva800 where two or three receptors were needed for a virus to gain entry into the cell both gave optimal values for the efficiency of the delivery virus equal to that found for Tva950. Of these models, that in which two receptors were needed for viral entry gave a better fit to the data (lowest model error in Table 1).

## Discussion

There has been considerable recent interest in understanding the stoichiometric aspects of viral binding and entry; however, previous studies have tended to concentrate on the stoichiometry of viral envelope binding in relation to entry [1-4]. Theoretical models presented here show how low surface receptor expression could also affect viral binding and entry. Relating the models shown in Figure 6 to the experimental data presented here would suggest that binding of more than one Tva800, probably two, by a virus is necessary in order for the virus to enter the cell, but that binding of only one Tva950 is sufficient. It must be emphasized that our study should not be taken as suggesting a physiological role for AGG mediated translation; rather, we have taken advantage of this observation to study normal receptor requirements for virus uptake.

Our study started with a chance observation related to poor efficiency of Tva800 receptor transfer and presumably stems from a random PCR error affecting the translational start site of this gene. We showed that *Mus dunni *cells alone are non-permissive to infection mediated by ASLV Env, but the introduction of the Tva800 AGG vector renders them permissive to infection. It had previously been shown that expression of low levels of either Tva800 or Tva950 protein is both necessary and sufficient for infection by ASLV-A Env pseudotyped virions [11]. Therefore, after the transduction of MDTF cells with the Tva800AGG vector, functional Tva800 protein must be expressed at some level on the cell surface. This requires in-frame initiation of protein synthesis downstream of the transcription start site, but upstream of the Tva800 signal sequence required for protein sorting, which is predicted to lie in the N-terminal 19 amino acids of Tva [11], without an intervening translation stop site. To examine this issue, we sequenced the whole Tva800 AGG vector. The (DNA) sequence of the transcript encoding Tva800, from transcriptional start to the translation stop at the end of the ORF, is shown in Additional file 2. The Tva800 ORF, including its AGG start site, is shown in blue. All ATGs upstream of the Tva800 ORF are highlighted in red, and stop codons in-frame with the ORF are underlined. Examination of this sequence reveals an opal stop codon 60 nucleotides upstream of the Tva800 ORF with no intervening AUG. Alternatively, RNA splicing might juxtapose an in frame ATG with the ORF. Indeed in silico analysis [25] revealed that the T to G alteration in the normal start codon yields a possible splice acceptor site. However, the only upstream splice donor predicted was the normal Mo-MLV splice donor at position 223 (GenBank Accession Number AFO33811) and there are no ATG triplets before this. We therefore conclude that the Tva800 ORF is translated using a non-canonical start codon. Such start sites are not unknown, but are more usually found in plant genomes, bacteria, yeast, and viruses, and initiation is much weaker than from AUG [26-31]. There is, however, an in frame CTG triplet, the most commonly used alternative start codon in mammals [28,31], between -61 and the normal start site. The vectors Tva950AGG and Tva800AGG are identical in their backbones, including the promoter and intergenic regions, and only differ near the 3' end of the Tva ORF hence the potential start site of Tva950AGG must be the same as Tva800AGG. Irrespective of the exact start site, translation of the Tva800AGG and Tva950AGG is likely to be inefficient. Nevertheless, given the need for a leader peptide, it seems likely that the structure of the expressed ectodomain seen by virus will be the same as with wild type receptor.

Application of a mathematical model gave an understanding of the physical processes underlying the observed behaviour of the cells. Optimising the parameters of the model to fit the experimental results suggested a differing mode of viral entry to the cell via the Tva800 and Tva950 receptors. The receptor efficiency for Tva800 was high, at close to 100%, while the receptor efficiency for Tva950 was low, at around 20%. It must be considered how similar or different really are the mechanisms of the two receptors, Tva800 and Tva950. A comparison of the graphs in Figures 3 (Tva800) and 5 (Tva950) with the theoretical models shown in Figure 6 would suggest that there is a significant difference between the two. The two forms of the receptor differ only in their attachment to the membrane and the localised milieu of the plasma membrane (Tva800 resides in lipid rafts) [12]. Tva950 is associated with faster rates of internalization, probably due to a faster rate of endocytosis and lateral diffusion of the receptor over the cell membrane [17,18]. Tva800 did not appear to increase the rate of endocytosis over non-specific levels, possibly because as a GPI-anchored receptor lacks the intracellular domain that can participate in signalling and recruitment of endocytic apparatus. Plasma membrane proteins attached via a GPI anchor are only associated with the outer leaflet of the plasma membrane and it is possible that the perturbation to the cell membrane necessary for fusion of viral and cellular membranes, and hence viral entry, is more readily achieved when the receptor spans both leaflets, as is the case for Tva950. It is also possible that, since Tva800 is not anchored to both leaflets, it is more prone to being extruded from the cell membrane due to the force exerted on it subsequent to virus binding, in comparison to Tva950.

One possibility not explicitly included in the model is that Tva800 and Tva950 might exist in clusters so that incoming viruses hit more than one receptor at once. If Tva950 is found in clusters, the apparent result that only one receptor is needed for entry into the cell could arise from the simultaneous binding of multiple receptors, as a virus interacts with a single cluster (the mathematical model does not differentiate between a single receptor, or a cluster of multiple receptors). For Tva800, however, whether the virus initially binds to a cluster or a receptor, there is a clear requirement for further binding to free clusters or free receptors that must take place before entry, shown by the decrease in infection when high titres of virus are used. Whether receptors occur in isolation, as assumed by the model, or in clusters, the same qualitative result, of the virus needing to bind multiple Tva800 receptors/clusters as opposed to a single Tva950 receptor/cluster stands, though the precise number of receptors required would be quantitatively different if clusters formed in significant numbers.

Overall, the data presented here show that low levels of receptor expression can prevent high titres of virus from being able to enter cells if the virus needs to bind more than one receptor in order to do so. Studying these effects is important for applications involving gene therapy in clinical settings. Traditionally it has been assumed that a high viral titre is necessary in order for entry of proportions sufficient to achieve the desired effect [32,33]. However, this assumption and strategy could be counter-productive if viral envelopes and receptor combinations with entry requirements such as ASLV Env and Tva800 are used, as excessive numbers of virions would actually inhibit entry rather than increase titres.

## Conclusions

We show that viral entry via Tva950 requires one receptor, and via Tva800 requires more than one receptor, probably two. The difference is due to the two modes of attachment to the receptors to the cell membrane. The simplicity of the ASLV Env, Tva800 and Tva950 system, without additional factors or co-receptors, lends itself as an advantageous model to facilitate further studies in the area of viral entry.

## Methods

### Cells and viruses

*Mus dunni *tail fibroblast (MDTF) and 293T cells were cultivated in Dulbecco modified Eagle medium supplemented with 10% fetal calf serum and antibiotics. Viruses were generated by transient transfection of 293T cells with three plasmids providing vector, *gag-pol*, and *env *by a conventional CaPO_4 _method as described previously [34]. Briefly, 1 day before transfection, 293T cells were seeded at 2 × 10^6 ^cells in 5 ml culture medium on 6-cm-diameter dishes. For preparing the "delivery" vector viruses, 7 μg each of pVSV-G [34], pHIT60 (MLV *gag-pol*) [35], and delivery vector plasmids were transfected simultaneously. For preparing ASLV-A-pseudotyped NB-MLV "tester" viruses, which carry enhanced green fluorescent protein (EGFP), 293T cells were transfected with 7 μg each of pLNCG [21], pHIT60 and pCB6-EnvA, [36]. Medium containing SUA-rIgG was prepared by transfection of 293T 10 μg of the plasmid pSUA-rIgG (a gift of John Young) [37] as for tester viruses. At 18 h after transfection, cells were treated with 10 mM sodium butyrate for 6 h to stimulate cytomegalovirus promoter-driven expression. At 48 h after transfection, the virus-containing culture supernatant was harvested, filtered through a 0.45 μm-pore-size filter, and stored immediately at -80°C. Titres of viral stocks were established by FACS.

### Generation of Tva containing plasmids

Plasmids expressing Tva800 and Tva950 [11] were gifts from John Young. The products of amplification of Tva800 and Tva950 (nucleotides 41-405 and 41-554 of GenBank Accession Numbers L22752 and L22753 respectively) were cloned into pLgatewayIY [21] resulting in MoMLV LTR-promoter driven Tva and IRES-YFP vectors using methods described in [34]. Plasmids Tva800AGG and Tva950ATG were generated in this fashion with the T to G change in Tva800AGG presumably resulting from an error during PCR. Mutation of Tva800AGG to Tva800ATG, and Tva950ATG to Tva950AGG was done by PCR site-directed mutagenesis using PfuUltra DNA polymerase (Stratagene) according to the manufacturer's protocol with the following oligonucleotides. Tva800AGG; forward, CCGCCCCCTTCACCATGGAGAGGATGATGCC and reverse, GGCAGCAGCCGCGCCATGGTGAAGGGGGCGG. Tva950ATG; forward CCCCCTTCACCAGGGAGAGGATGCTG and reverse, CAGCAGCCGCGCCCTGGTGAAGGGGG. The nine amino acid HA-tag was inserted into plasmids between amino acids 23 and 24 of Tva800 and Tva950 by insertional PCR using the following oligonucleotides. Forward, TACCCTTACGATGTTCCTGATTACGCTAACGGGTCCGGTAACGGTTCTTTGTCCCG and reverse CGCGTAATCAGGAACATCGTAAGGGTAACCGGTCACGTTACCGGGCAGC. The final, mutated sequences were verified by sequencing.

### Assay of Tva dependent entry

Assays for entry were carried out as described previously for Fv1 restriction assays [34]. Briefly, 5 × 10^4 ^MDTF cells per well were seeded on a 12-well plate. Sixteen hours later, cells were challenged with delivery vector virus to transduce the Tva800ATG, Tva800AGG, Tva950ATG or Tva950AGG construct and EYFP. Approximately 48 h later, cells were split 1:12, and after a further 16 h, they were challenged with ASLV-A Env-pseudotyped tester virus to transduce EGFP. Cells were inoculated with aliquots of delivery virus at MOIs (a ratio of the number of infectious units per target cell, defined by measurement of virus on a permissive cell line) of less than 1 to over 20 infectious units/cell, and tester virus levels of between 1-600 μl, or < 1-99% green cells in control cells, which were MDTF cells transduced with pLgateway800IG which constitutively express the Tva800 receptor. When cells were infected with tester virus in competition with SUA-rIgG, the viral dose was maintained at 10 μl with 0-500 μl SUA-rIgG-containing medium, made up to 510 μl in PBS. Forty-eight hours after the second transduction, cells were harvested, fixed in phosphate-buffered saline (PBS)-3.5% formalin, and examined for EGFP and EYFP expression by fluorescence-activated cell sorting analysis with a fluorescence-activated cell sorter LSR apparatus (Becton Dickinson). Titres of virus were assessed by calculating the percentage of GFP-positive cells in the YFP-positive cell populations.

### Western blot

MDTF cells transduced at different MOI of the Tva800ATG or Tva800AGG constructs were seeded in 60 mm plates and grown to confluence. The cells were washed with PBS twice, and lysed in 500 μl 1xSDS loading buffer (50 mM Tris-HCl, 100 mM DTT, 2% SDS, 0.1% Bromophenol Blue, 10% glycerol) at 95°C, and removed from the plates using a cell scraper. The samples were boiled for 10 minutes, cleared by centrifugation, and separated by polyacrylamide gel electrophoresis. Proteins were then transferred onto a polyvinylidene difluoride membrane, and incubated overnight in blocking buffer (PBS containing 5% milk and 0.1% Tween 20) at 4°C overnight. The membrane was incubated in blocking buffer containing polyclonal anti-HA (Sigma) (1 in 5,000 dilution) or anti-α-tubulin (Sigma) (1 in 10,000 dilution) antibody at room temperature for 1 hour. After the membrane was washed, it was incubated at room temperature for 1 hour in blocking buffer containing horseradish peroxidase-conjugated anti-rabbit immunoglobulin G antibody (1 in 20,000 dilution) for HA detection or horseradish peroxidase-conjugated protein A (1 in 10,000 dilution) for α-tubulin detection. After the membrane was washed, the protein bands were detected using the enhanced chemiluminescence (ECL) system (Amersham).

### Mathematical modelling of viral entry

A statistical model was applied to calculate the theoretical percentage of MDTF cells expressing EYFP that also expressed EGFP. The values for the MOIs of the delivery vector virus and the tester virus that were used in the experiment were taken as inputs to the model, and five parameters were used to describe the physical behaviour of the system. Describing the interaction with the delivery vector virus, the efficiency of the delivery vector, *p*, was defined as the probability that an interaction of a cell with a delivery vector virus would lead to the expression of Tva, while the number of receptors produced by a cell per delivery provirus was described by the parameter *r*. Describing the interaction of the tester virus with receptors on the cell, the number of receptors a virus needed to bind to gain entry to the cell was described by the parameter *k*, while the receptor efficiency *q *was defined as the probability that the binding of a virus to *k *receptors would lead to the expression of EGFP. For systems in which tester viruses needed to bind more than one receptor to gain entry into the cell, a final parameter, *γ*, described the ratio between the rate at which viruses in solution bound to receptors on the cell, and the rate at which viruses already bound to receptors on the cell surface acquired more receptors. For selected values of *k*, the other parameters were optimised through a computational process to give the best fit to the experimental data. Full details of the model are given in additional file 1.

## Competing interests

The authors declare that they have no competing interests.

## Authors' contributions

ERG carried out the molecular and virological analyses and drafted the mauscript. CJRI refined the model and performed the mathematical analyses. JMC originally suggested the modelling approach and helped revise the manuscript. JPS conceived the study and participated in its design and helped draft the manuscript. All authors read and approved the final manuscript.

## Supplementary Material

Additional file 1 **Details of the mathematical model**. A description of the derivation of a statistical model to calculate the percentage of MDTF cells expressing EYFP that also expressed EGFP. Click here for file

Additional file 2 **Partial sequence of the Tva800AGG vector from R-U5 to the end of the Tva800 ORF**. Sequence of a region of plasmid Tva800AGG. Stop codons in-frame with the Tva800 ORF have been underlined, ATG codons are marked in red, and the Tva800 ORF is in blue. Click here for file

## References

[B1] Magnus C Rusert P Bonhoeffer S Trkola A Regoes RR Estimating the stoichiometry of human immunodeficiency virus entry J Virol 2009 83 1523 1531 10.1128/JVI.01764-08 19019953 PMC2620894

[B2] Klasse PJ Modeling how many envelope glycoprotein trimers per virion participate in human immunodeficiency virus infectivity and its neutralization by antibody Virology 2007 369 245 262 10.1016/j.virol.2007.06.044 17825343 PMC2317823

[B3] Yang X Kurteva S Lee S Sodroski J Stoichiometry of antibody neutralization of human immunodeficiency virus type 1 J Virol 2005 79 3500 3508 10.1128/JVI.79.6.3500-3508.2005 15731244 PMC1075697

[B4] Yang X Kurteva S Ren X Lee S Sodroski J Stoichiometry of envelope glycoprotein trimers in the entry of human immunodeficiency virus type 1 J Virol 2005 79 12132 12147 10.1128/JVI.79.19.12132-12147.2005 16160141 PMC1211524

[B5] Yang X Kurteva S Ren X Lee S Sodroski J Subunit stoichiometry of human immunodeficiency virus type 1 envelope glycoprotein trimers during virus entry into host cells J Virol 2006 80 4388 4395 10.1128/JVI.80.9.4388-4395.2006 16611898 PMC1472027

[B6] Alkhatib G Combadiere C Broder CC Feng Y Kennedy PE Murphy PM Berger EA CC CKR5: a RANTES, MIP-1alpha, MIP-1beta receptor as a fusion cofactor for macrophage-tropic HIV-1 Science 1996 272 1955 1958 10.1126/science.272.5270.1955 8658171

[B7] Deng H Liu R Ellmeier W Choe S Unutmaz D Burkhart M Di Marzio P Marmon S Sutton RE Hill CM Identification of a major co-receptor for primary isolates of HIV-1 Nature 1996 381 661 666 10.1038/381661a0 8649511

[B8] Dragic T Litwin V Allaway GP Martin SR Huang Y Nagashima KA Cayanan C Maddon PJ Koup RA Moore JP Paxton WA HIV-1 entry into CD4+ cells is mediated by the chemokine receptor CC-CKR-5 Nature 1996 381 667 673 10.1038/381667a0 8649512

[B9] Feng Y Broder CC Kennedy PE Berger EA HIV-1 entry cofactor: functional cDNA cloning of a seven-transmembrane, G protein-coupled receptor Science 1996 272 872 877 10.1126/science.272.5263.872 8629022

[B10] Young JA Bates P Varmus HE Isolation of a chicken gene that confers susceptibility to infection by subgroup A avian leukosis and sarcoma viruses J Virol 1993 67 1811 1816 8383211 10.1128/jvi.67.4.1811-1816.1993 PMC240233

[B11] Bates P Young JA Varmus HE A receptor for subgroup A Rous sarcoma virus is related to the low density lipoprotein receptor Cell 1993 74 1043 1051 10.1016/0092-8674(93)90726-7 8402880

[B12] Narayan S Barnard RJ Young JA Two retroviral entry pathways distinguished by lipid raft association of the viral receptor and differences in viral infectivity J Virol 2003 77 1977 1983 10.1128/JVI.77.3.1977-1983.2003 12525631 PMC140899

[B13] Mothes W Boerger AL Narayan S Cunningham JM Young JA Retroviral entry mediated by receptor priming and low pH triggering of an envelope glycoprotein Cell 2000 103 679 689 10.1016/S0092-8674(00)00170-7 11106737

[B14] Barnard RJ Narayan S Dornadula G Miller MD Young JA Low pH is required for avian sarcoma and leukosis virus Env-dependent viral penetration into the cytosol and not for viral uncoating J Virol 2004 78 10433 10441 10.1128/JVI.78.19.10433-10441.2004 15367609 PMC516436

[B15] Barnard RJ Elleder D Young JA Avian sarcoma and leukosis virus-receptor interactions: from classical genetics to novel insights into virus-cell membrane fusion Virology 2006 344 25 29 10.1016/j.virol.2005.09.021 16364732

[B16] Delos SE Brecher MB Chen Z Melder DC Federspiel MJ White JM Cysteines flanking the internal fusion peptide are required for the avian sarcoma/leukosis virus glycoprotein to mediate the lipid mixing stage of fusion with high efficiency Journal of Virology 2008 82 3131 3134 10.1128/JVI.02266-07 18184714 PMC2259008

[B17] Lim KI Narayan S Young JA Yin J Effects of lipid rafts on dynamics of retroviral entry and trafficking: Quantitative analysis Biotechnol Bioeng 2004 86 650 660 10.1002/bit.20108 15137076

[B18] Jha NK Latinovic O Martin E Novitskiy G Marin M Miyauchi K Naughton J Young JA Melikyan GB Imaging single retrovirus entry through alternative receptor isoforms and intermediates of virus-endosome fusion PLoS Pathog 2011 7 e1001260 10.1371/journal.ppat.1001260 21283788 PMC3024281

[B19] Stremlau M Owens CM Perron MJ Kiessling M Autissler P Sodroski J The cytoplasmic body component TRIM5α restricts HIV-1 infection in Old World monkeys Nature 2004 427 848 853 10.1038/nature02343 14985764

[B20] Best S Le Tissier P Towers G Stoye JP Positional cloning of the mouse retrovirus restriction gene *Fv1* Nature 1996 382 826 829 10.1038/382826a0 8752279

[B21] Yap MW Nisole S Lynch C Stoye JP Trim5alpha protein restricts both HIV-1 and murine leukemia virus Proc Natl Acad Sci USA 2004 101 10786 10791 10.1073/pnas.0402876101 15249690 PMC490012

[B22] Mitchell H Levin D Forrest S Beauchemin CA Tipper J Knight J Donart N Layton RC Pyles J Gao P Higher level of replication efficiency of 2009 (H1N1) pandemic influenza virus than those of seasonal and avian strains: kinetics from epithelial cell culture and computational modeling J Virol 2011 85 1125 1135 10.1128/JVI.01722-10 21068247 PMC3019989

[B23] Kadolsky UD Asquith B Quantifying the impact of human immunodeficiency virus-1 escape from cytotoxic T-lymphocytes PLoS Comput Biol 2010 6 e1000981 10.1371/journal.pcbi.1000981 21079675 PMC2973816

[B24] McKinley TJ Murcia PR Gog JR Varela M Wood JL A Bayesian approach to analyse genetic variation within RNA viral populations PLoS Comput Biol 2011 7 e1002027 10.1371/journal.pcbi.1002027 21483482 PMC3068928

[B25] Brunak S Engelbrecht J Knudsen S Prediction of human mRNA donor and acceptor sites from the DNA sequence J Mol Biol 1991 220 49 65 10.1016/0022-2836(91)90380-O 2067018

[B26] Gren EJ Recognition of messenger RNA during translational initiation in Escherichia coli Biochimie 1984 66 1 29 10.1016/0300-9084(84)90188-3 6370317

[B27] Kozak M Comparison of initiation of protein synthesis in procaryotes, eucaryotes, and organelles Microbiol Rev 1983 47 1 45 6343825 10.1128/mr.47.1.1-45.1983 PMC281560

[B28] Depeiges A Degroote F Espagnol MC Picard G Translation initiation by non-AUG codons in Arabidopsis thaliana transgenic plants Plant Cell Rep 2006 25 55 61 10.1007/s00299-005-0034-0 16184386

[B29] Cherpillod P Zipperle L Wittek R Zurbriggen A An mRNA region of the canine distemper virus fusion protein gene lacking AUG codons can promote protein expression Arch Virol 2004 149 1971 1983 15669108 10.1007/s00705-004-0342-7

[B30] Chen SJ Lin G Chang KJ Yeh LS Wang CC Translational efficiency of a non-AUG initiation codon is significantly affected by its sequence context in yeast J Biol Chem 2008 283 3173 3180 18065417 10.1074/jbc.M706968200

[B31] Wegrzyn JL Drudge TM Valafar F Hook V Bioinformatic analyses of mammalian 5'-UTR sequence properties of mRNAs predicts alternative translation initiation sites BMC Bioinformatics 2008 9 232 10.1186/1471-2105-9-232 18466625 PMC2396638

[B32] Burns JC Friedmann T Driever W Burrascano M Yee JK Vesicular stomatitis virus G glycoprotein pseudotyped retroviral vectors: concentration to very high titer and efficient gene transfer into mammalian and nonmammalian cells Proceedings of the National Academy of Sciences of the USA 1993 90 8033 8037 10.1073/pnas.90.17.8033 8396259 PMC47282

[B33] Zhang J Frolov I Russell SJ Gene therapy for malignant glioma using Sindbis vectors expressing a fusogenic membrane glycoprotein The journal of gene medicine 2004 6 1082 1091 10.1002/jgm.605 15368589

[B34] Bock M Bishop KN Towers G Stoye JP Use of a transient assay for studying the genetic determinants of Fv1 restriction J Virol 2000 74 7422 7430 10.1128/JVI.74.16.7422-7430.2000 10906195 PMC112262

[B35] Soneoka Y Cannon PM Ramsdale EE Griffiths JC Romano G Kingsman SM Kingsman AJ A transient three-plasmid expression system for the production of high titer retroviral vectors Nucleic Acids Res 1995 23 628 633 10.1093/nar/23.4.628 7899083 PMC306730

[B36] Gilbert JM Bates P Varmus HE White JM The receptor for the subgroup A avian leukosis-sarcoma viruses binds to subgroup A but not to subgroup C envelope glycoprotein J Virol 1994 68 5623 5628 8057442 10.1128/jvi.68.9.5623-5628.1994 PMC236963

[B37] Zingler K Young JA Residue Trp-48 of Tva is critical for viral entry but not for high-affinity binding to the SU glycoprotein of subgroup A avian leukosis and sarcoma viruses J Virol 1996 70 7510 7516 8892869 10.1128/jvi.70.11.7510-7516.1996 PMC190818

