# Supplementary material for: Binding of more than one Tva800 molecule is required for ASLV-A entry
Source: Retrovirology. 2011 Nov 18;8:96. doi: 10.1186/1742-4690-8-96 (PMC3267798; doi:10.1186/1742-4690-8-96)
Supplement: Additional file 1 — Details of the mathematical model. A description of the derivation of a statistical model to calculate the percentage of MDTF cells expressing EYFP that also expressed EGFP. [file 1742-4690-8-96-S1.DOC]

**Additional file 1**

**Details of the mathematical model**

A statistical model was applied to calculate the theoretical percentage of MDTF cells expressing EYFP that also expressed EGFP. Interactions of the cells with the delivery vector virus were modelled using a Poisson process. Where *m* was the MOI with which the MDTF cells were inoculated with the delivery vector virus, the interaction between cells and viruses was modelled as a Poisson process, such that the probability of a cell interacting with *v* viruses was given by

$$P\left(k in teractions\right)=\frac{e^{-m}m^{v}}{v!}$$

Here it was assumed that an interaction with the delivery vector virus would inevitably lead to the cell expressing EYFP. We let *p* be the probability that an interaction will also result in the expression of Tva by the cell. Assuming viral interactions with the cell to be independent, the probability that there will be *n* expressions of Tva in a cell that interacts with *v* viruses was calculated using a binomial distribution. Where *^v^C_n_* is the binomial coefficient

$${}^{v}C_{n}=\frac{v!}{n!\left(v-n\right)!}$$

$$P\left(\left.TVA ex pressed n times\right|v in teractions\right)={}^{v}C_{n}p^{n}\left(1-p\right)^{v-n}$$

and summing over possible values of *v*, the probability *p_n_* of a cell expressing Tva *n* times given at least one interaction with the delivery vector virus was calculated using the expression

$$p_{n}=\sum_{v=1}^{\infty} \frac{e^{-m}m^{v}{}^{v}C_{n}p^{n}\left(1-p\right)^{v-n}}{v!}$$

In the model, therefore, cells expressing YFP have a distribution of levels of Tva integration, with frequencies described by the values *p_n_*.

Considering the interaction of cells with the tester virus, it was assumed that each integration of Tva leads to the expression of a fixed number of receptors, *r*. To allow non-integer values of *r*, giving flexibility to the model, the probability $P_{R}\left(i\right)$ that an integration of Tva would lead to the expression of *i* receptors was defined as

$$P_{R}\left(i\right)=\left\{\begin{matrix} 0 \\ 1-\left(r-\left\lfloor r\right\rfloor\right) \\ r-\left\lfloor r\right\rfloor\end{matrix}\right. \begin{matrix} i<r, i>r+1 \\ i=r \\ i=r+1 \end{matrix}$$

where $\left\lfloor r\right\rfloor$ is the greatest integer less than or equal to *r*. The expected number, *R*, of receptors expressed by the cell population as a whole was then calculated as

$$R=\sum_{n=1}^{\infty} p_{n}nr$$

In the experimental work, different quantities of solution containing the tester virus were added to the cells, leading in each case to some MOI of tester viruses, *M*. Where *M* was the number of viruses per cell, the expected value of *V*, the number of viruses per receptor in the population, was calculated as

$$V=M/R$$

We next considered an arbitrary cell in the system with *n* Tva expressions, under the condition that the delivery virus needed to bind to *k* receptors in order to gain entry into the cell. Under the model above, the cell would have *nr* receptors, such that the initial interaction of viruses with receptors could be described by a Poisson process with rate *nrV*. In contrast to the interaction with delivery vector viruses, however, the rate of the process would not be constant, with binding of viruses to receptors reducing the number of receptors available to other viruses. The binding of viruses to the cell was, therefore, modelled as a more general continuous time Markov process, in which the rate of interaction of new viruses with the cell decreased in proportion to the number of receptors remaining. Supposing that at some arbitrary time, the number of viruses bound to *i* receptors was equal to *n_i_*, the number of receptors still available for binding, *b*, was calculated as

$$b=nr-\sum_{i=1}^{k} in_{i}$$

Under this model, the rate at which new viruses bind to the cell is equal to *bV*, where *b* depends on the current state of the cell.

To model the process in which viruses already bound to receptors on the cell acquire additional receptors, it was assumed that the inherent rate of acquisition of new receptors by receptor-bound viruses moving within the membrane could be related to the inherent rate at which viruses in solution bind to receptors by means of a constant ratio *γ*, independent of the number of receptors already bound, with *γ* >1 implying that viruses bound to receptors bind additional receptors more quickly than do viruses in solution. Assuming that at some arbitrary time there are *n_i_* viruses each bound to *i* receptors, the corresponding rate of increase in *n_i+1_* was calculated as *γn_i_b*, for all 0≤*i*<*k*

Given the above model, the probability that a cell expressed GFP was then calculated. The probability that at some time *t* a cell has *n_i_* viruses bound to *i* receptors was denoted by

$P\left(n_{1},...,n_{k}\right)_{t}$

At time *t*=0 no viruses were bound to the receptors on the cell, such that

$P\left(0,...,0\right)_{0}=1$

As a full analytic solution of the continuous time Markov process would be difficult, a discrete time approximation was applied. Considering a small timestep ε, changes in the distribution of bound receptors were calculated using the expression

$\begin{matrix} P\left(n_{1},...,n_{k}\right)_{t+\varepsilon}=H\left(n_{1}-1\right)\left[1-\exp\left(-V\varepsilon\left(b+1\right)\right)\right]P\left(n_{1}-1,n_{2},...n_{k}\right)_{t} \\ +\sum_{j=1}^{k-1} \left(H\left(n_{j+1}-1\right)\left[1-\exp\left(-\gamma\varepsilon\left(n_{j}+1\right)\left(b+1\right)/N\right)\right]P\left(n_{1},...,n_{j}+1,n_{j+1}-1,...,n_{k}\right)_{t}\right) \\ +\left[\exp\left(-V\varepsilon b\right)-\sum_{j=1}^{k-1} \left(H\left(n_{j}\right)\left[1-\exp\left(-\gamma\varepsilon n_{j}b/N\right)\right]\right)\right]P\left(n_{1},...,n_{k}\right)_{t} \end{matrix}$

where the first two terms on the RHS of the equation represent the influx of viruses from solution and the acquisition of additional receptors by viruses bound in the cell membrane, and

$$H\left(x\right)=\left\{\begin{matrix} 0, \\ 1, \end{matrix}\right. \begin{matrix} x<0 \\ x\geq0 \end{matrix}$$

is the Heaviside step function. As ε→0, the model tends towards an exact solution.

Given a numerical solution of the model, we supposed that the probability of a single virus gaining entry to the cell leading to the expression of GFP was equal to some value, *q*. The probability of a cell expressing GFP was then calculated as:

$$\sum_{n_{k}=1}^{\left\lfloor nr/k\right\rfloor} \left[\left(1-\left(1-q\right)^{n_{k}}\right)\sum_{n_{1},n_{2},...,n_{k-1}} P\left(n_{1},...,n_{k}\right)_{1}\right]$$

where $\left\lfloor x\right\rfloor$ is the greatest integer less than or equal to *x*.

This model, which describes a cell with *nr* receptors, was extended to cover the entire population of cells, using the probabilities *p_n_* derived above. For cells in the entire population, the probability of a cell that expressed EYFP also expressing EGFP was given by

$$\sum_{n=1}^{\infty} p_{n}\left[\sum_{n_{k}=1}^{\left\lfloor nr/k\right\rfloor} \left(\left(1-\left(1-q\right)^{n_{k}}\right)\sum_{n_{1},n_{2},...,n_{k-1}} P\left(n_{1},...,n_{k}\right)_{1}\right)\right]$$

For computational reasons the sum to infinity was truncated, being calculated only over values of *n* for which *p_n_* was at least 10^-10^ times the size of the maximum value of *p_n_*.

**Parameter estimation**

Parameters for the MOI of the MDTF and tester viruses used in the experimental work were input into the model, the resulting values {*m_ij_*} for the fraction of YFP cells expressing GFP being compared to the experimental values {*e_ij_*}. For each chosen value of *k*, the remaining parameters (*p*, *r*, *q*, and where appropriate, *γ*) were optimized so as to minimise the difference between the model and experimental results, this difference being calculated using an RMSD score:

$$D\left(p,r,q,\gamma\right)=\sqrt{\sum_{i,j} \left(m_{ij}-e_{ij}\right)^{2}}$$

Optimization was carried out using a grid based method, adding ±*dx* to each of the parameters, retaining changed parameters where doing so reduced the value of *D*, and reducing *dx* when a local minima was found. This process was repeated until a set of parameters was found that constituted a minima at all values of *dx* (values for *dx* being taken from the set [0.2, 0.1, 0.05, 0.02, 0.01, 0.005, 0.002, 0.001]). Qualitatively the function *D* had the appearance of being globally convex with the addition of a small amount of noise, suggesting the minima found to be the global minimum for the system.

The value of *ε* was set to 5x10^-4^. In the models of Tva800 with *k*=1 and *k*=2, and in the model of Tva950, the RMSD difference between the respective model values {*m_ij_*} for *ε*=10^-3^ and *ε*=5x10^-4^ was less than 1% of the value of *D*.
