# Supplementary material for: Binding of more than one Tva800 molecule is required for ASLV-A entry
Source: Retrovirology. 2011 Nov 18;8:96. doi: 10.1186/1742-4690-8-96 (PMC3267798; doi:10.1186/1742-4690-8-96)
Supplement: Additional file 2 — Partial sequence of the Tva800AGG vector from R-U5 to the end of the Tva800 ORF. Sequence of a region of plasmid Tva800AGG. Stop codons in-frame with the Tva800 ORF have been underlined, ATG codons are marked in red, and the Tva800 ORF is in blue. [file 1742-4690-8-96-S2.DOC]

**Additional file 2**

**Partial sequence of the Tva800AGG vector from R-U5 to the end of the Tva800 ORF**

G CGC CAG TCC TCC GAT TGA CTG AGT CGC CCG GGT ACC CGT GTA TCC AAT AAA CCC TCT TGC AGT TGC ATC CGA CTT GTG GTC TCG CTG TTC CTT GGG AGG GTC TCC TCT GAG TGA TTG ACT ACC CGT CAG CGG GGG TCT TTC ATT TGG GGG CTC GTC CGG GAT CGG GAG ACC CCT GCC CAG GGA CCA CCG ACC CAC CAC CGG GAG GTA AGC TGG CCA GCA ACT TAT CTG TGT CTG TCC GAT TGT CTA GTG TCT ATG ACT GAT TTT ATG CGC CTG CGT CGG TAC TAG TTA GCT AAC TAG CTC TGT ATC TGG CGG ACC CGT GGT GGA ACT GAC GAG TTC GGA ACA CCC GGC CGC AAC CCT GGG AGA CGT CCC AGG GAC TTC GGG GGC CGT TTT TGT GGC CCG ACC TGA GTC CTA AAA TCC CGA TCG TTT AGG ACT CTT TGG TGC ACC CCC CTT AGA GGA GGG ATA TGT GGT TCT GGT AGG AGA CGA GAA CCT AAA ACA GTT CCC GCC TCC GTC TGA ATT TTT GCT TTC GGT TTG GGA CCG AAG CCG CGC CGC GCG TCT TGT CTG CTG CAG CAT CGT TCT GTG TTG TCT CTG TCT GAC TGT GTT TCT GTA TTT GTC TGA AAA TAT GGG CCC GGG CTA GCC TGT TAC CAC TCC CTT AAG TTT GAC CTT AGG TCA CTG GAA AGA TGT CGA GCG GAT CGC TCA CAA CCA GTC GGT AGA TGT CAA GAA GAG ACG TTG GGT TAC CTT CTG CTC TGC AGA ATG GCC AAC CTT TAA CGT CGG ATG GCC GCG AGA CGG CAC CTT TAA CCG AGA CCT CAT CAC CCA GGT TAA GAT CAA GGT CTT TTC ACC TGG CCC GCA TGG ACA CCC AGA CCA GGT CCC CTA CAT CGT GAC CTG GGA AGC CTT GGC TTT TGA CCC CCC TCC CTG GGT CAA GCC CTT TGT ACA CCC TAA GCC TCC GCC TCC TCT TCC TCC ATC CGC CCC GTC TCT CCC CCT TGA ACC TCC TCG TTC GAC CCC GCC TCG ATC CTC CCT TTA TCC AGC CCT CAC TCC TTC TCT AGG CGC CCC CAT ATG GCC ATA TGA GAT CTG TCG AAT CAC AAG TTT GTA CAA AAA AGC AGG CTC CGC GGC CGC CCC CTT CAC AGG GCG CGG CTG CTG CCC GCG CTG CTG CTG CTG CTG CTG CCC GGT AAC GTG ACC GGT AAC GGG TCC GGT AAC GGT TCT TTG TCC CGT TGC CCC CCC GGT CAG TTC CGC TGC TCG GAG CCG CCC GGT GCC CAC GGG GAG TGT TAC CCG CAG GAC TGG CTG TGC GAC GGA CAC CCC GAC TGC GAC GAC GGG CGG GAC GAG TGG GGC TGC GGG ACC AGC GCG ACC CCC GCG GTG CCC ACG GAC AAC GGC ACA GAG GCT CCC ACT GTC CCT GCT CCT GGA CGT GCT CTG CCA GCC AGG AAT CAC GGC CGC ATG TGG ATG CTG ATC ACT GCA GGG ATC TTT TGC TGT GAG CTG GTG AGA TGG GAC TGA

Supplementary figure 1. Partial sequence of the Tva800AGG vector from R-U5 to the end of the Tva800 ORF. Stop codons in-frame with the Tva800 ORF have been underlined, ATG codons are marked in red, and the Tva800 ORF is in blue.
